# Public management attitudes and behavioural intentions towards the management of (over)abundant wild ungulate populations

**DOI:** 10.1007/s13280-025-02258-x

**Published:** 2025-10-23

**Authors:** Antonio J. Carpio, Roberto Pascual-Rico, Tamara Murillo-Jiménez, María Martínez-Jauregui, Rafael Villafuerte-Jordán, Pelayo Acevedo

**Affiliations:** 1 Instituto de Investigación en Recursos Cinegéticos, IREC (UCLM-CSIC-JCCM), Ronda Toledo 12, 13071 Ciudad Real, Spain; 2 Área de Ecología, Departamento de Botánica, Ecología y Fisiología Vegetal, Universidad de Córdoba, Campus Universitario de Rabanales, 14014 Córdoba, Spain; 3 Department of Education, Faculty of Educational Sciences and Psicology, Universidad de Córdoba, 14071 Córdoba, Spain; 4 Instituto de Ciencias Forestales (ICIFOR, INIA-CSIC), Ctra. De La Coruña km. 7.5, 28040 Madrid, Spain; 5 Departamento de Biología Aplicada, Universidad Miguel Hernández de Elche, Av. de la Universidad S/N, 03202 Elche, Spain

**Keywords:** Control measures, Stewardship, Tolerance, Ungulate management, Wildlife acceptance capacity

## Abstract

Social perceptions of wild ungulates depend on their abundance and the socioeconomic context. These perceptions shape attitudes toward management and explain social behavior toward the species. Our objective was to evaluate attitudes and behavioral intentions toward wild ungulate management in the Iberian Peninsula. We conducted a survey of 440 participants in mainland Spain. Respondents generally supported non-lethal methods, though attitudes varied with socioeconomic context. A key finding was that attitudes did not directly translate into behavioral intentions, suggesting the influence of cognitive dissonance or social norms. While many respondents supported education and awareness actions, a considerable share reported no intention to participate or provide financial support. Recreational hunting was broadly rejected. Notably, hunters’ involvement in management was associated with attitudes that more strongly predicted behavioral intentions. These results highlight the complexity of the social landscape and underscore the need to adapt management strategies to socioeconomic settings and behavioral drivers.

**Supplementary Information:**

The online version contains supplementary material available at 10.1007/s13280-025-02258-x.

## Introduction

Interest in managing ungulate populations has increased due to their ecological and socio-economic impacts, such as biodiversity loss, habitat degradation, and agricultural damage (Apollonio et al. 2010; Carpio et al. 2021; Pascual-Rico et al. 2021; Pringle et al. 2023). Their widespread distribution across anthropized European landscapes has led to diverse human-wildlife interactions, creating challenges for effective population management (Apollonio et al. 2017; Linnell et al. 2020).

Public attitudes and actions towards ungulate management vary depending on the species and socio-economic context (Liordos et al. 2017; Martínez-Jauregui et al. 2020, 2023). For instance, rural farmers affected by wild boar (*Sus scrofa*) damage may support population control, while others prefer indirect measures to mitigate damage (Kansky et al. 2014). This reflects a broader challenge in ungulate management aimed to reconcile agricultural interests with conservation goals, especially when damage control measures are socially or politically contested. Similarly, despite broad agreement on controlling overabundant ungulate populations in National Parks, stakeholder opposition delayed management, worsening ecosystem damage (e.g. Perea et al. 2015; Martínez-Jauregui et al., 2021). This case highlights the difficulty of implementing timely management actions when public perception and legal constraints hinder necessary interventions, a recurring issue in protected areas worldwide. These examples illustrate the complex trade-offs that wildlife managers must navigate, balancing ecological, economic, and social considerations when designing effective ungulate management policies.

Recent studies indicate that ungulates, such as red deer (*Cervus elaphus*), are generally well perceived and not widely considered problematic across different socio-economic contexts (Valente et al. 2020; 2024; Carpio et al. 2024). This perception persists even in areas where ungulates cause environmental or economic damage, suggesting a discrepancy between societal attitudes and the necessity for management interventions (Martínez-Jauregui et al. 2023). Understanding societal perceptions and attitudes towards wild ungulates is crucial for designing effective management strategies (Cebrián-Piqueras et al. 2023; Martínez-Jauregui et al. 2023). Perception refers to how people interpret ungulates and their management. Attitudes regarding population management reflect support for specific control measures (Ajzen 1991). The Theory of Planned Behaviour (TPB; Ajzen 1991) provides a useful framework to explain how attitudes, social norms, and perceived behavioural control influence public support or opposition to ungulate management measures. In this context, factors such as past experiences, self-identity (e.g. hunters vs. conservationists), and emotional responses shape opinions on interventions, such as hunting, translocation, or indirect control strategies (Conner and Armitage 1998).

Behavioural intentions towards ungulate management indicate the likelihood of engaging in management actions; therefore, they are not solely based on attitudes but are influenced by contextual willingness to act. While some individuals actively support population control, others prefer minimal intervention, highlighting the importance of management strategies that align with public perceptions (Valente et al. 2020). Recognizing these behavioural drivers is essential for implementing effective, publicly supported conservation policies. In some cases, society supports population control financially, through direct contributions or public funding allocations, reflecting their stance on wildlife management (Martínez-Jauregui et al. 2020; Van Eeden et al. 2021; Gross et al. 2021). Others engage in volunteer activities, such as population monitoring or habitat restoration, demonstrating a personal commitment to wildlife (Comte et al. 2022). These actions often stem from personal values and emotional connections to nature, aligning with the TPB, where identity and social norms influence engagement in conservation efforts.

Some citizens may not engage in ungulate management due to lack of interest, insufficient knowledge, or the belief that it is not their responsibility (Keuling et al. 2016; Carpio et al. 2024). This is especially true in areas where ungulates are not perceived as a major issue or where conflicts do not directly affect human activities (Kansky and Knight 2014). Analysing these behaviours through the lens of the TPB can provide insights into the feasibility of different management actions and the factors shaping public engagement. Such an approach is essential for developing effective and socially acceptable management strategies that account for diverse societal perspectives (Honda et al. 2018).

The present study was designed to address the issue of society’s support for ungulate population management, responding to critical knowledge gaps regarding how public attitudes and behavioural intentions shape the acceptance of different management strategies. Although prior research has explored the ecological and economic impacts of ungulate populations, fewer studies have examined how socio-economic contexts influence support for management actions, particularly in Southern European landscapes where ungulate-human interactions are increasingly complex. Understanding these social dimensions is essential for designing effective, publicly supported management strategies (Cebrián-Piqueras et al. 2023; Martínez-Jauregui et al. 2023). In this context, the specific objectives of this study were to: (i) quantify the level of societal management attitudes regarding specific wild ungulate management measures (positive or negative agreement with management actions) in both general and different socio-economic contexts; (ii) describe society’s behavioural intentions towards the management of wild ungulate species in both general and specific socioeconomic contexts; and (iii) establish the potential relationships between behavioural intentions and attitudes towards ungulate management. The study focuses on wild ungulate species inhabiting the Iberian Peninsula, a region where expanding populations of ungulates pose new ecological and socio-political challenges (Carvalho et al. 2025). By integrating ecological and social perspectives, this research fills an important gap in conservation planning, providing insights that can inform adaptive, context-specific wildlife management policies.

## Materials and methods

### Design of questionnaire

In order to contextualize the questions related to management attitudes and behavioural intentions, we considered various management strategies for ungulate populations in different ecological, social and economic contexts. The selection of these strategies was based on common applied methods in wild ungulate population control (e.g. Pascual-Rico et al. 2021). These strategies encompassed both direct control methods, such as recreational hunting, culling, and translocation, as well as indirect strategies, including fencing, deterrents, predator promotion, and garbage control. The aim was to ensure a comprehensive assessment of public preferences about the management across different territorial contexts (urban, agricultural, forest, livestock, hunting, and protected areas).

The questionnaire was ad hoc and designed to align with the study’s objectives, assessing public attitudes towards different wild ungulate management strategies and behavioural intentions regarding their implementation across socio-economic contexts. The questions were structured to capture the level of agreement with management actions, willingness to contribute (financially or voluntarily), and potential barriers to engagement. It constructed based on a thorough literature review and the study objectives (Annex 1). The development process consisted of four steps (see also Carpio et al. 2024): (i) selecting and formulating items based on the reviewed literature; (ii) content validation through expert evaluation (n = 10; see Lawshe 1975; Tristán-López 2008); (iii) comprehension validation via a pilot study with 40 respondents to assess understandability; and (iv) reliability analysis using internal consistency testing (Cronbach’s Alpha During content validation), experts rated each item based on pertinence, relevance, and clarity, using a four-point Likert scale (4 = a lot, 3 = quite a lot, 2 = a little, 1 = none). Items with a content validity ratio (CVR) above 0.58 were retained, ensuring expert consensus on their adequacy. The final content validity index (CVI) confirmed the robustness of the instrument for assessing societal attitudes and behavioural intentions (Annex 2a). The questionnaire and its implementation were approved by the Research Ethics Committee of the University of Cordoba (Spain), reference number 5091.

The questionnaire consisted of three well-differentiated sections, the first of which had the objective of obtaining socio-demographic information about the respondents, such as their gender, age group, academic qualifications, and the interest group/s to which they belonged (hunters, farmers, livestock breeders, ecologists, or the general public). The second section focussed on evaluating the respondents’ attitudes towards management and the contexts in which they found ungulate management measures most appropriate (objective i). The respondents were asked about the extent to which they agreed with various measures used to control ungulate populations, including *management hunting, recreational hunting, live capture and slaughter, translocations, contraception, promotion of natural predator populations,* and *garbage control* (Table 1). The respondents were also asked about their preferred measures (only one) in different contexts, such as agriculture, urban areas, forestry, protected areas, hunting, or livestock farms. The questions used in this section were based on a five-point Likert scale from one to five *(totally disagree; disagree; neutral; agree; totally agree)* (Annex 1, where *neutral* response may reflect both indecision due to lack of knowledge and a balanced view where respondents acknowledge both benefits and drawbacks of population control measures). The objective of the third section was to gain insights into the respondents’ behavioural intentions regarding ungulate overabundance (objective ii). This part of the questionnaire explored the actions that respondents would be willing to take in order to address the ungulate self-reported overabundance (Carpio et al. 2024). The respondents were specifically asked whether they would be open to making financial donations, participating as volunteers, or not contributing in any way in order to address the issue (Table 1). The respondents were also invited to prioritize what they considered to be the most important action as regards controlling ungulate population size within each context and for each species (the aoudad *Ammotragus lervia*, the chamois *Rupicapra pyrenaica*, the fallow deer *Dama dama*, the Iberian wild goat *Capra pyrenaica*, the red deer, the roe deer *Capreolus capreolus*, the mouflon *Ovis orientalis musimon*, and the wild boar). Finally, two specific variables were obtained: management attitudes (composed of nine items) and behavioural intentions (made up of 12 items) (Table 1). Thus, to analyse the relationship between attitudes and behavioural intentions will allow us to reach the last objective (objective iii).

**Table 1:** List of items corresponding to management attitudes (n = 9) and behavioural intentions towards ungulate management (n = 12).* I would be willing to make a financial donation so that, in my province…** I would be willing to collaborate as a volunteer through education or awareness actions and report on my social networks in order to… ***I would not contribute in any way to changing this situation because…

ATTITTUDES		BEHAVIOURAL INTENTIONS	
Item	Statement	Item	Statement
A1	**Management hunting**: Environmental agents must control the size of populations	B1	*…environmental agents carry out population **control programmes** (management hunting, live capture)
A2	**Hunter-managed hunting**: Hunters, under the supervision of environmental officers, help to control population sizes	B2	*…**recreational hunting** is encouraged
A3	**Recreational hunting**: Hunters directly manage populations	B3	*…**other alternatives** are encouraged: increase in predators, contraception, fencing, supplementary feeding…
A4	**Live capture**, animal handling and on-site **slaughter**	B4	*… environmental agents would be **prevented** from carrying out population **control programmes** (management hunting, live capture)
A5	Live capture, transport, and release to other locations (**translocation**)	B5	*…**recreational hunting** would **not** be encouraged
A6	**Contraception**: sterilization by mechanical or chemical means	B6	*…**other** population control **alternatives** would **not** be promoted: predator enhancement, contraception, fencing, supplementary feeding
A7	**Indirect measures**: Fencing of plants and crops, separation of livestock, traffic signs, etc	B7	**…promote **population control**
A8	Encouragement of populations of **natural predators**	B8	**…show **opposition to population control**
A9	Garbage control, **avoid additional** sources of **resources**…	B9	*** …the **problem** of ungulate abundance **is not sufficiently relevant** for me
		B10	*** …**others** should deal with this
		B11	*** …I believe that **no intervention in natural processes** should take place
		B12	***…other reasons

It is important to note that no items were reverse-coded. Opposing items (e.g. "promote population control" vs. "show opposition to population control") were treated as independent measures rather than being mathematically inverted. Thus, low agreement with an item supporting a specific action implies opposition, while high agreement with an item opposing that action indicates the same underlying stance. This approach ensures clarity and transparency in response interpretation.

During the questionnaire design we avoid the possibility of modifying previous answers, thus, we limited the possible bias due to respondents changed previous answers in order to be consistent throughout the questionnaire. However, this restriction may have also reduced respondents’ ability to refine or clarify their views as they progressed through the questionnaire, particularly when encountering new information or reconsidering their initial stance.

### Implementation of questionnaire

A sample of 440 individuals, who were representative of mainland Spanish society, participated in this study (Annex 2b). The participants were drawn from a consumer panel, which is a common practice in public preference research (e.g. Martínez-Jauregui et al. 2020). The entire convenience sample was used, without subset selection, ensuring that all participants contributed to the analysis. We acknowledge the inherent limitations associated with the use of consumer panels, since panels may sometimes be biased towards individuals with higher levels of education and technology use. To mitigate these biases, we stratified the sampling proportionally among participants according to socio-demographic characteristics (i.e. sex, age, study levels, residence). Besides, this methodology has been previously validated and applied in published research (Carpio et al. 2024). The online survey was conducted using the web platform^1^. Participation was voluntary, and no incentives were provided. All the participants provided informed consent before taking part in the study. It took the respondents an average of approximately 15 min to complete the questionnaire.

For minimizing potential uncertainties and biases in this convenience sampling method, several precautions and controls were implemented before data analysis (Maas et al. 2021). Firstly, the sample was stratified on the basis of age, gender, rural or urban populations, and regions, thus ensuring an appropriate proportion of respondents relative to the total population of mainland Spain (see Annex 3 for details). Second, reliability analyses of the scale were conducted using Cronbach’s Alpha, which indicated an internal consistency of above 0.75 for the dimension related to attitudes towards intervention measures and above 0.8 for the social behavioural intention dimension. These results have a good level of fit and provide confidence in the data quality (Cortina 1993).

### Statistical analysis

To analyse associations between agreement degree (based on a five-point Likert scale) related to management attitudes and behavioural intentions, we created contingency tables and performed Fisher’s exact test (α = 0.05). We applied Fisher exact test because expected frequencies from the contingency tables were less than 5, thereby ensuring statistical robustness. The same approach was used to assess the most preferred behavioural intentions across different contexts.

Relations between management attitudes and behavioural intentions were analysed by an adapted to categorical data Correspondence Analysis (CA; Greenacre 2013). The position of the points (attitudes and behavioural intentions) allows to know which attitudes and behavioural intentions are associated.

## Results

All respondents completed the full questionnaire, and they displayed varying levels of support for different ungulate management strategies, with values influenced by socio-economic contexts. Overall, there was a positive attitude towards indirect measures and controlled population management, while rejecting recreational hunting and invasive control methods (see Table 2). However, when asking about behavioural intentions respondents indicated that the most agreement item was with others should deal with this.

**Table 2:** Mean Likert value (from 1 to 5 for totally disagree to totally agree, respectively) and standard deviation (SD) for each management attitudes and behavioural intentions based on responses

Opinion on	Mean	SD
Management attitudes		
Management hunting	3.8	1.1
Hunter-managed hunting	3.3	1.6
Recreational hunting	2.1	1.1
Capture and slaughter	2.0	1.2
Translocation	3.3	1.3
Contraception	3.0	1.2
Indirect measures	3.9	1.0
Natural predation promoted	3.3	1.1
Garbage control	4.3	0.8
Behavioural intentions		
I would be willing to make a financial donation so that…		
Environmental agents control populations	2.6	1.3
Recreational hunting would be encouraged	1.8	1.0
Environmental agents would be prevented from population control	2.7	1.2
Other alternatives would be encouraged	2.3	1.2
Recreational hunting would not be encouraged	3.0	1.4
Other alternatives would not be encouraged	2.4	1.2
I would be willing to collaborate in order to…		
Promote population control	3.1	1.1
Opposite to population control	2.5	1.0
I would not contribute in any way because…		
The problem is not relevant	2.9	1.0
Others should deal with this	3.4	1.1
Interventions in natural processes should not take place	2.9	1.1

### Attitudes and behavioural intentions towards the management of abundance of ungulates populations

**Fig. 1:**
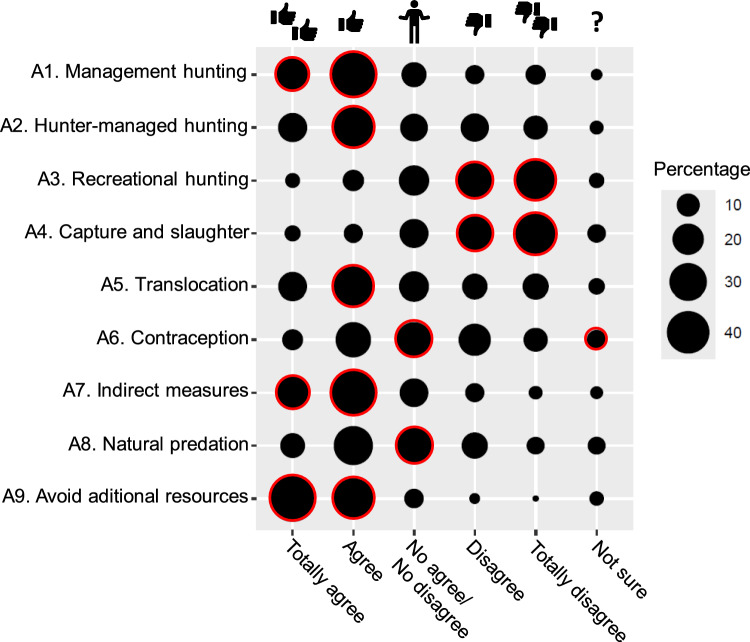
Management attitude responses (in percentage) on Likert-scale. Circles with red borders indicate that the management attitudes selected were higher than expected (*p* < 0.05)

Significant positive associations between attitudes towards particular management measures and agreement degree with them were found (χ^2^ = 1441.2, df = 40, *p* < 0.001; Fig. 1; Annex 4a). Our findings particularly revealed that the respondents significantly supported hunting as a management tool, even when carried out by hunters, but avoiding recreational hunting, capture and slaughter. The respondents also significantly agreed with other measures, such as translocations, indirect measures or avoiding additional resources. Measures as contraception and natural predation were significant selected as *No agree/No disagree*, and also *Not sure* for contraception.

**Fig. 2:**
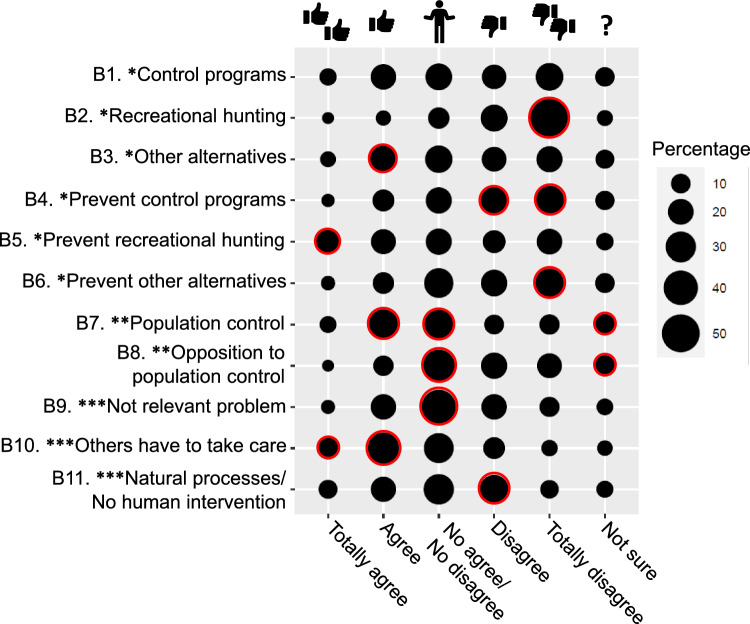
Behavioural intention responses (in percentage) on Likert-scale. Circles with red borders indicate that the intentions selected were higher than expected (*p* < 0.05).* I would be willing to make a financial donation so that, in my province… ** I would be willing to collaborate as a volunteer through education or awareness actions and report on my social networks in order to… ***I would not contribute in any way to changing this situation because…

We found positive associations between particular behavioural intentions and agreement with them (χ^2^ = 855.7, df = 50, *p* < 0.001; Fig. 2; Annex 4b). The respondents were significantly motivated to financially favour other alternatives, to financially avoid recreational hunting and to voluntarily collaborate in population control through education or awareness campaigns. The respondents also considered that others should deal with this situation (this is understood as *I would not contribute in any way to changing this situation*). Nevertheless, most responses were concentrated on point three of the Likert-scale (do not agree/do not disagree). This was particularly evident in items such as promoting education or awareness about *population control*, being in *opposition to population control*, and not contributing since *it is not a relevant problem.* Respondents also significant selected *Not sure* in the items promoting education or awareness about *population control*, being in *opposition to population control*.

### Influence of socio-economic context on management-related attitudes and behavioural intentions.

**Fig. 3:**
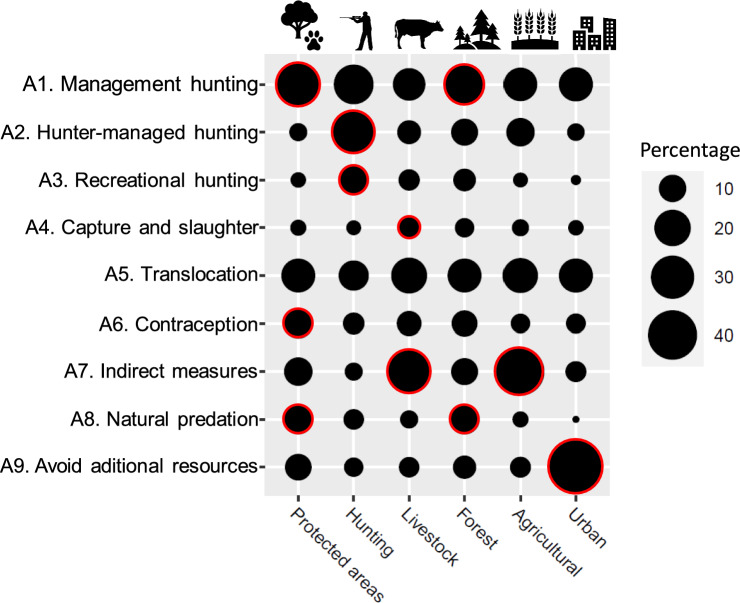
Relationship between management measure attitudes and overabundance contexts (N = 440 by context). Circles with red borders indicate that the management attitudes selected were higher than expected (*p* < 0.05)

With regard to contexts, different attitudes towards management measures were found (χ^2^ = 1296.6, df = 40, *p* < 0.001; Fig. 3; Annex 5a). In urban contexts, the respondents preferred management attitudes focussed on avoiding additional resources, whereas in agricultural and livestock contexts they preferred indirect management measures, along with capture and slaughter in a livestock context. In the case of forests and protected areas, people preferred management hunting as a management tool, in addition to natural predation, but also contraception in the case of protected areas. Finally, in hunting contexts, the respondents opted for hunter-managed hunting and recreational hunting as the main management measures to be applied.

With regard to behavioural intentions towards ungulate management and from a global point of view (i.e. regardless of the socioeconomic context), respondents chose mainly *no contribution options* (B9–B12), representing 36.4% of answers selected. Besides, they selected also mainly to financially support environmental agents carrying out population control programmes (20.6% of the respondents, B1), followed by financially *preventing recreational hunting* (B5, 11.7%) and the behavioural intention of collaborating as a volunteer through education or awareness actions in order *to promote population control* (B7, 10.6%).

**Fig. 4:**
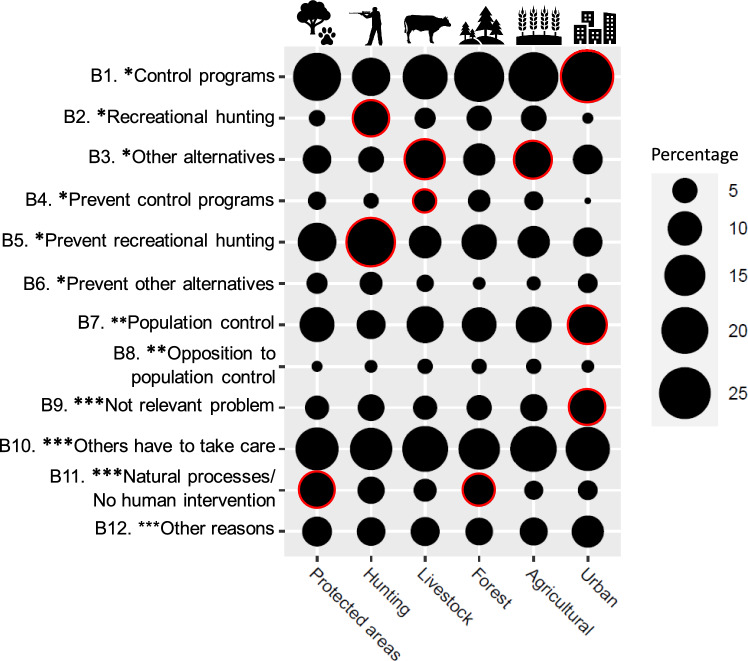
Relationship between behavioural intentions and overabundance contexts (N = 440 by context)*.* Circles with red borders indicate that the behavioural intentions selected were more common than expected (*p* < 0.05). * I would be willing to make a financial donation so that, in my province… ** I would be willing to collaborate as a volunteer through education or awareness actions and report on my social networks in order to… ***I would not contribute in any way to changing this situation because…

By contexts, we found significant support for particular behavioural intentions depending on the contexts (χ^2^ = 285.0, df = 55, *p* < 0.001; Fig. 4; Annex 5b). In that of protected and forest areas, no inhuman intervention stood out. In contrast, in that of livestock and agricultural areas, behavioural intentions such as financially promoting other alternatives or financially preventing environmental agents from carrying out population control programmes solely regarding the livestock context were statistically highlighted. In the hunting context, there were opposite attitudes regarding behavioural intentions related to recreational hunting, signifying that it was deemed that recreational hunting should be both financially encouraged and financially prevented. Finally, in the urban context, behavioural intentions related to financially promoting population control stood out (*financially supporting control programs*; *voluntarily collaborating on population control*), but this was also considered not to be a relevant problem for the respondents.

### Relationship between management attitudes and behavioural intentions

**Fig. 5:**
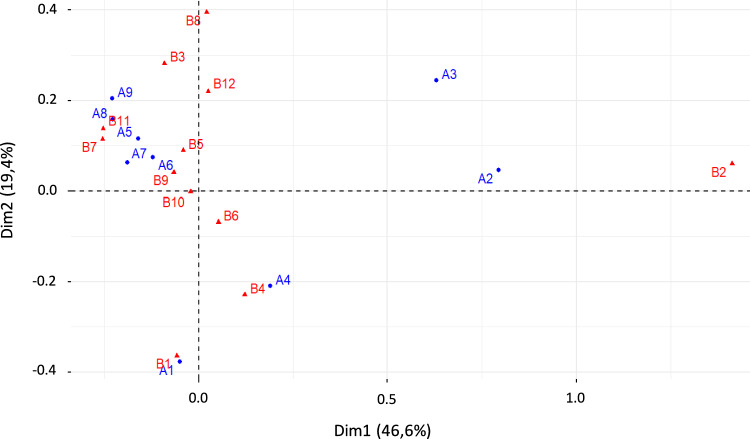
Correspondence analysis performed on the attitudes (blue) and behavioural intentions (red). Attitudes: A1. Management hunting; A2. Hunter-managed hunting; A3. Recreational hunting; A4. Capture and slaughter; A5. Translocation; A6. Contraception. A7; Indirect measures; A8. Natural predation; A9. Avoid additional resources. Behaviours: B1. Control programs; B2. Recreational hunting; B3.Other alternatives; B4.Prevent control programs; B5.Prevent recreational hunting; B6.Prevent other alternatives; B7. Population control; B8. Opposition to population control; B9. Not relevant problem; B10. Others have to take care; B11. Natural processes/No human intervention; B12 Other reasons

The Correspondence Analysis performed explained 66.6% of the total variation, considering the first two axes. Figure 5 shows associations between particular management attitudes and behavioural intentions. Particularly the attitudes *hunters-managed hunting* (A2) and *recreational hunting* (A3) are closer to the behavioural intention *willing to donate for recreational hunting* (B2). These three variables are the ones that contribute most to the axis 1. Moreover, the attitude *management hunting by agents* (A1) and the behaviour *to donate for control programs by agents* (B1) are closer, and therefore, related to. Attitudes on lethal control of ungulates where hunters collaborate (A2 and A3) are the most relevant and differentiating to explain the variability in behavioural intentions, especially highlighting their link with B2.

**Fig. 6:**
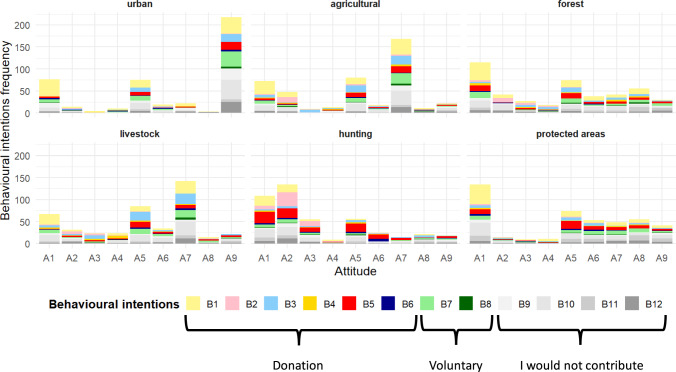
Interrelations between behavioural intentions and management attitudes preferred by respondents by each context (Urban, Agricultural, Forest, Livestock, Hunting and Protected areas)

We additionally analysed the relationship between management attitudes and behavioural intentions by considering the socio-economic context (Fig. 6; Annex 6). It stands out that among the most selected behaviours highlight *no contribution options* (B9-B12) and *to donate for control programs by agents* (B1). Besides, there is a high variability among different behavioural intentions for a given attitude among contexts. For instance, in the urban contexts, the attitude to *avoid additional resources* (A9) is the most selected by respondents that support *no contribution options*, *to donate for control programs by agents* and *to collaborate on population control* (B7). With regard to agricultural and livestock contexts, most people selected *indirect measures* (A7), and respondents showed different behavioural intentions as *no contribution options* (B9-B12), *to donate for control programs by agents* (B1) and *to promote other alternatives* (B3). In hunting areas, the main management attitude was *hunter-managed hunting* (A2), followed by *management hunting by agents* (A1), however, the main behavioural intention shown here was to *donate to prevent recreational hunting*.

## Discussion

Overall, the most frequently supported management attitudes emphasized controlled interventions, such as regulated hunting and translocation, alongside indirect strategies that minimize direct human intervention, such as avoiding additional resources for ungulates. Besides, most of the participants expressed neutrality towards certain behavioural intentions, they tended to favour financial support for alternative management approaches, opposition to recreational hunting, and participation in educational and awareness initiatives. Additionally, a portion of respondents expressed a preference for non-involvement, indicating that they believed responsibility for population control should fall on other entities. Interestingly, the results obtained also show how management attitudes and social behavioural intentions change depending on the socio-economic context (Steinwall 2015; Martinez-Jauregui et al. 2020).

### Attitudes and behavioural intentions towards management of abundance of ungulates populations

Overall, societal attitudes towards ungulate management measures indicate a general preference for non-lethal methods, such as translocation (A5), indirect measures (A7) or avoiding additional resources (A9). However, our results also suggest that management hunting is accepted when used specifically as a population control tool rather than for recreational purposes (A1 and A2). This distinction highlights an important differentiation between ethical concerns, which drive opposition to recreational hunting (A3) and practical considerations, which lead to the acceptance of targeted lethal methods aimed at mitigating ecological or economic impacts.

Despite the inherent costs and complexity that translocations entail, this method is generally preferred by society (McCann et al. 2021). However, translocation poses ethical concerns, as it can disrupt recipient ecosystems and cause long-term consequences (Harrington et al. 2022). Similarly, indirect measures are among the most frequently selected management attitudes, and are explained by moral considerations (Fischer et al. 2013). These moral considerations generally include a preference for non-intrusive methods that avoid causing direct harm to animals, reflecting a concern for animal welfare and ethical treatment (Decker et al. 2012). For example, measures such as habitat modifications (i.e. fences) or the use of deterrents are perceived as being more humane when compared to lethal methods. The emphasis on indirect measures aligns with broader ethical values that prioritize reducing direct harm and suffering, and maintaining an ecological balance. This preference for non-lethal methods reflects a societal inclination to minimize direct harm to wildlife, while also acknowledging the need to balance ethical considerations with practical management challenges (Martínez-Jauregui et al. 2020).

Moreover, it has been shown that lethal methods gain acceptance when such actions effectively mitigate impacts related to ungulate population size (Hare et al. 2021), and when the purpose, associated impacts and risks are transparently communicated to society (Martinez-Jauregui and Soliño 2021). The acceptance of lethal methods, therefore, largely depends on the motivation behind their implementation (i.e. recreational hunting, population control, or management hunting; Garrido et al. 2017; Gamborg et al. 2018). In this respect, hunting and management plans should be clearly presented as population control tool rather recreational activities to enhance public support.

Respondents generally expressed financial support for alternative methods that avoid lethal population control and indicated a willingness to collaborate as volunteers in education and awareness initiatives related to population management. However, recreational hunting was broadly rejected, as it is often perceived as ethically contentious (Szklarska 2020). Besides respondents showed agreement with idea that management should be handled by others (B10), but they also showed disagreement with non-intervention (B11). This suggests that while respondents recognize the need for action, they prefer to delegate responsibility rather than engage directly. This confirms that stated by Martinez-Jauregui et al. (2020), which showed that only 2% of respondents thought that national parks should have no human intervention, even when a lack of management can lead to environmental degradation. The tendency to assign responsibility to external entities has been previously observed in specific social groups, such as hunters, many of whom perceive ungulate management as a task for authorities rather than their own responsibility (Keuling et al. 2016).

### Influence of socio-economic context on management-related attitudes and behavioural intentions.

Management attitudes and behavioural intentions differ depending on each particular socio-economic context. In protected and forest areas, although respondents supported low-intervention strategies for population control as a management attitude (A1, A6 and A8), as other authors pointed (Porter 1992), their behavioural intentions leaned towards allowing natural processes to regulate populations rather than direct intervention (B11). The issue of intervening in nature in order to maintain or restore values is highly contested and complex, as it often involves ethical, environmental and social considerations (Steinwall 2015). This perception arises because society often views protected areas or forests as pristine ecosystems, reflecting the western idea of a separation between pristine nature and human-modified habitats (Valbuena-Carabaña et al. 2010). However, many hunting grounds are located in forests, thus public perception tends to associate forests with natural conservation areas, leading to lower support for recreational hunting also in these contexts. Nevertheless, active human intervention in these areas should not be seen as a threat but as a necessary substitute for traditional ecological processes, such as grazing, fires, and natural disturbances (Bugalho et al. 2011; Sebek et al. 2015).

With regard to the hunting context (generally overlapping with forest context in our study area), the management attitudes supported were hunter-managed hunting (A2) and recreational hunting (A3), although this support varied depending on the hunting motivation (Gamborg et al. 2018; Garrido et al. 2017). This could explain society’s polarization towards both behaviours (willing to pay for (B2) and against (B5) recreational hunting) even in hunting areas (Gamborg and Jensen 2017), as the behavioural intentions related to recreational hunting selected were the opposite. This discrepancy likely stems from the distinction between recreational hunting, which is often viewed as a sport-driven activity, and management hunting, which is implemented with clear ecological objectives. Consequently, recreational hunting is often viewed as incompatible with conservation principles, even in areas where hunting is a legally sanctioned activity.

In the agricultural and livestock contexts, the measures that most stand out among management attitudes are those of indirect management (A7), which includes fencing to protect crops or to separate livestock from wildlife. This is further reinforced by the findings on behavioural intentions, which reflect respondents’ willingness to financially support alternative measures (B3). The preference for these strategies may be linked to economic and health concerns, as farmers and livestock breeders face direct financial losses due to crop damage and disease transmission from wild ungulates (Martínez-Jauregui et al. 2020; Widén et al. 2023). Although fences may have the potential to eliminate damage, their installation and maintenance can be expensive (Walter et al. 2010). The alignment between management attitudes and behavioural intentions in these contexts suggests that economic incentives or compensation schemes could enhance the acceptance of population control measures.

In the urban context, the most frequently selected management attitude was that of avoiding anthropogenic food availability (A9, i.e. garbage control). This could be related to the impossibility of employing lethal measures that could suppose a risk to humans (e.g. hunting), and has been shown to be an efficient strategy (González-Crespo et al. 2018). With regard to behavioural intentions, responses were polarized: while some respondents supported population control programs (B1 and B7), others expressed indifference (B9–B12), likely because they had not experienced direct encounters or conflicts with ungulates. This aligns with previous studies indicating that individuals who have faced urban wildlife-related issues—such as traffic accidents, damage to gardens, or aggressive encounters—tend to support control measures, whereas those with no personal experience often perceive the issue as irrelevant (Conejero et al. 2019). This finding suggests that awareness campaigns could be effective in increasing public understanding of urban wildlife management challenges. These differences have important policy implications. In urban contexts, public support for financial contributions and education campaigns indicates a preference for indirect engagement in management efforts. Wildlife managers could implement awareness programs to improve public understanding of the ecological impact of ungulates in urban environments and promote preventive measures such as waste management policies and restrictions on wildlife feeding to limit food availability.

### Relationship between management attitudes and behavioural intentions

According to our results the participation of one of the considered main stakeholder in wild ungulate management (i.e. hunters) influences on attitudes and behavioural intentions of the society. This recreational hunting opposition is also evident among different contexts, what reflects the previous commented social polarization regarding recreational hunting (Gamborg and Jensen 2017).

Many participants remained neutral regarding certain behavioural intentions, which may reflect different underlying attitudes. Some respondents may be apathetic, showing little concern or engagement with ungulate management. Others might be sceptical, questioning the effectiveness or necessity of intervention measures. Additionally, some may perceive ungulate overabundance as not an urgent issue, particularly if they have not directly experienced conflicts with these species. Despite that, this neutrality was not reflected on attitudes towards management measures, as a high percentage tended to take a clear stance either agreeing or disagreeing depending on each particular management measure. This information is particularly relevant for informing management decisions, as individuals may disagree with certain measures or hold negative attitudes towards them, yet take no further action or shift responsibility to others. In such cases, their opposition tends to have less impact on management processes and may not directly constrain decision-making. This suggests that while individuals may have clear opinions on whether a management strategy is appropriate, they may hesitate when it comes to actively engaging in or financially supporting these measures. This distinction is crucial for wildlife management policies, as it highlights the gap between public support for strategies in principle and their willingness to take action (Veríssimo and Campbell 2015).

Therefore, our results showed that behavioural intentions are not fully explained by attitudes towards management measures alone, because other factors, such as social norms and perceived behavioural control, may add information that drives the final decision, what aligns with the TPB (Ajzen 1991; Conner and Armitage 1998). Cognitive factors, such as attitudes towards specific behaviours and perceived behavioural control, play a crucial role. For instance, if individuals perceive that they have sufficient financial resources and trust that their contribution will be effectively used, their intention to donate increases. Social norms also shape behavioural intentions, as individuals tend to align their decisions with the perceived expectations of their social environment (Chung and Rimal 2016). Moreover, self-identity and personal values influence these decisions, as those who strongly identify with ecological responsibility are more inclined to support wildlife management measures. Beyond cognitive and social influences, practical constraints also impact behavioural choices. Previous experiences, availability of resources and opportunities, such as participation in volunteer programs, can reinforce future engagement, particularly if past experiences were positive (Tomkovick et al. 2008). Recognizing these interconnected factors is essential for designing management strategies that align with public motivations, ultimately increasing support and participation in conservation initiatives.

From a global point of view and in particular socio-economic contexts, the respondents were consistent as regards relating management attitudes and preferred behavioural intentions. This signifies that those respondents who considered it relevant to make a payment about population control carried out by environmental agents (B1) had positive attitudes towards game management (A1). The same occurred with those who supported no intervention (B11), which was related to management measures focussed on the promotion of predators (A8) and avoiding access to human resources (A9). However, behavioural intentions varied depending on the socio-economic context. In urban areas, lower exposure to ungulate-related conflicts could explain why respondents prefer to not contribute (B9–B12) and to place responsibility on authorities. In contrast, in hunting and forest areas, cultural norms seem to shape this response, as people may perceive that hunters or conservation managers should be primarily responsible for population control (Kaltenborn et al. 2006; Gamborg and Jensen 2017). These differences suggest that attitudes towards responsibility delegation are context-dependent, shaped by economic, experiential, and cultural factors.

This study has significant implications for both sociology and ungulate management. Policy frameworks should remain adaptable, allowing adjustments based on shifting public perceptions Veríssimo and ecological needs. These insights provide a foundation for evidence-based conservation policies, ensuring that ungulate management strategies are not only ecologically effective but also socially accepted.

## Conclusions

Not all attitudes are directly reflected in behaviour intentions, in many cases there is a lack of engagement from society, which should be taken into account by managers. The reasons behind this no-engagement are diverse, such as cognitive factors, social norms, personal values and even experiences. The most relevant attitudes for explaining behaviour are those related to hunters’ involvement in the implemented management measures. Moreover, the attitudes and behaviours preferred by society vary across different socio-economic contexts, which should also be considered in the decision-making process.

## Supplementary Information

Below is the link to the electronic supplementary material.

Supplementary file1 (PDF 587 KB)

## Footnotes

1
www.tickstat.com.
