# Supplementary material for: Public management attitudes and behavioural intentions towards the management of (over)abundant wild ungulate populations
Source: Ambio. 2025 Oct 23;55(3):647–60. doi: 10.1007/s13280-025-02258-x (PMC12868418; doi:10.1007/s13280-025-02258-x)

Ambio

Supplementary Information

This supplementary information has not been peer reviewed.

Title: **Public management attitudes and behavioural intentions towards the management of (over)abundant wild ungulate populations**

ANNEX 1 - Questionnaire on society's perceptions of wild ungulates in mainland Spain.

Municipality of habitual residence:	Date:
Gender:	Year of birth (before 1956; 1957-1966; 1967-1976; 1977-1986; 1987-1996; 1997-2003):
Level of studies "completed" (none; Primary school; Secondary school; University; PhD):	
Are you in any of the following situations? (You can mark several options):<br>- Member of Association linked to nature conservation<br>- Work related to the natural environment<br>- Small game hunter<br>- Big game hunter<br>- Farmer<br>- Rancher<br>- Owner of land exceeding 50 ha<br>- None of the above	

2- MANAGEMENT ATTITUDES

What is your opinion of the most common forms of intervention in situations in which animals (ungulates) cause unacceptable damage, where 1 is totally disagree and 5 is totally agree?

	1	2	3	4	5	I'm not sure
Management hunting: Environmental agents must control the size of populations						
Hunter-managed hunting: Hunters, under the supervision of environmental officers, help to control population sizes						
Recreational hunting: Hunters directly manage populations						
Live capture, animal handling and on-site slaughter						
Live capture, animal handling, transport and release to other locations						
Contraception: sterilization by mechanical or chemical means.						
Indirect measures: Fencing of plants and crops, separation of livestock, traffic signs, etc.						
Encouragement of populations of natural predators						
Garbage control, avoiding additional sources of resources, …						

Which is the most suitable for you in each zone?

	Urban	Agricultural	Forest	Livestock	Hunting	Protected
Management hunting: Environmental agents must control the size of populations						
Hunter-managed hunting: Hunters, under the supervision of environmental officers, help to control population sizes						
Recreational hunting: Hunters directly manage populations						
Live capture, animal handling and on-site slaughter						
Live capture, animal handling, transport and release to other locations						
Contraception: sterilization by mechanical or chemical means.						
Indirect measures: Fencing of plants and crops, separation of livestock, traffic signs, etc.						
Encouragement of populations of natural predators						
Garbage control, avoiding additional sources of resources, …						

3- BEHAVIOURAL INTENTIONS

To what extent do you agree with the following statements, where 1 is totally disagree and 5 is totally agree?

**I would be willing to make a financial donation so that, in my province...**	1	2	3	4	5	I'm not sure
…environmental agents could carry out population control programmes (management hunting, live capture)						
…recreational hunting would be encouraged						
…other alternatives would be encouraged: increase in predators, contraception, habitat management, control of supplementary feeding, …						
…environmental agents would be prevented from carrying out population control programmes (management hunting, live capture)						
…recreational hunting would not be encouraged						
…other population control alternatives would not be promoted: predator enhancement, contraception, habitat management, supplemental feeding control						
**I would be willing to collaborate as a volunteer through education or awareness actions and report on my social networks in order to…**						
…promote population control						

…show opposition to population control						
**I would not contribute in any way to changing this situation because...**						
... the problem of ungulate abundance is not sufficiently relevant for me						
... others should deal with this						
... I believe that interventions in natural processes should not take place						

Of the measures mentioned above, which is your preferred measure based on the territory in which it takes place?

**I would be willing to make a financial donation so that, in my province...**	Urban	Agricultural	Forest	Livestock	Hunting	Protected
…environmental agents could carry out population control programmes (management hunting, live capture)						
…recreational hunting would be encouraged						
…other alternatives would be encouraged: increase in predators, contraception, habitat management, control of supplementary feeding, …						
…environmental agents would be prevented from carrying out population control programmes (management hunting, live capture)						
…recreational hunting would not be encouraged						
…other population control alternatives would not promote: predator enhancement, contraception, habitat …management, supplemental feeding control						
**I would be willing to collaborate as a volunteer through education or awareness actions and report on my social networks in order to…**						
…promote population control						
…show opposition to population control						
**I would not contribute in any way to changing this situation because...**						
... the problem of ungulate abundance is not sufficiently relevant for me						
... others should deal with this						
... I believe that interventions in natural processes should not take place						
Other reasons						

What is your favourite measurement for each species present in your province? (Please write the number that corresponds)

**I would be willing to make a financial donation so that, in my province...**	Aoudad	Iberian ibex	Red deer	Roe deer	Fallow deer	Wild boar	Mouflon	Chamois
…environmental agents carry out population control programs (management hunting, live capture)								
…recreational hunting is encouraged								
…other alternatives are encouraged: increase in predators, contraception, habitat management, control of supplementary feeding, …								
…prevent environmental agents from carrying out population control programs (management hunting, live capture)								
…prevent recreational hunting from being encouraged								
…prevent other population control alternatives from being promoted: predator enhancement, contraception, habitat management, supplemental feeding control								
**I would be willing to collaborate as a volunteer through education or awareness actions and report on my social networks to…**								
…promote population control								
…show opposition to population control								
**I would not contribute in any way to change this situation because...**								
... the problem of ungulate abundance is not relevant enough to me								
... others have to take care of this								
... I believe that natural processes should not be intervened								
Others reasons								

ANNEX 2a - Content validation.

With respect to the questionnaire, i) content validity was carried out by means of expert opinion (Galicia Alarcón et al., 2017). The method implemented is based on that of Muñoz-Repiso et al. (2020). In this process, 10 experts were contacted who provided us with a reliable estimation of content validity (Hyrkäs et al., 2003). Each item was assessed according to three characteristics: pertinence, relevance and clarity, which were defined as follows:

1. Pertinence: the degree to which the indicator could measure competence.
2. Relevance: the suitability of the indicator as regards the level of competence.
3. Clarity: the suitability of the wording of the indicator (does not lead to different interpretations).

A four-point Likert scale was used (4-a lot, 3-quite a lot, 2-a little and 1-none), and each item also had a space in which to add comments. This allowed the experts to incorporate their suggestions regarding a particular item (see Muñoz-Repiso et al., 2020 for more details). Finally, the expert assessments and the level of agreement were processed following the method proposed by Lawshe (1975) and the review by Tristán-López (2008). The minimum value of the agreement is a constant proportion of the number of judges when standardizing the statistic CVR´, where a consensus of at least 58.23% must be reached in order for an item to be accepted. After the CVR values have been obtained for each item, it is possible to determine the CVI (Content Validity Index) of an instrument or bank of items, which in our case is the indicator model.

Annex 2 shows the sum of the CVR (content validity ratio) included in the model, the number of items that obtained a positive relationship from the experts (M) and whether or not the items were accepted. The level of adequacy of the sociodemographic data, the level of adequacy of the items concerning behavioural intentions, and management attitudes towards wild ungulates in Spain (dimensions 1 and 2) are acceptable, since the satisfaction indicator provided by the experts was > 0.5823. All of the questions in Section 1 were deemed essential; however, one question was modified in accordance with the experts' comments.

Table S1. Content Validity Indexes of the initial model (Muñoz-Repiso et al., 2020).

Level of adequacy of…	N° of items	N° of experts	Evaluated characteristics	Σ items (CVR´)	M	Acceptable	Partial CVI	Total CVI
**socio-demographic data items**	5	10	Essential	4.65	5	Yes	0.93	0.93
**management attitude items**	9	10	Clarity	8.6	9	Yes	0.95	0.98
			Pertinence	9	9	Yes	1	
			Relevance	9	9	Yes	01	
**behavioural intention items**	14	10	Clarity	13.2	14	Yes	0.94	0.95
			Pertinence	13.4	14	Yes	0.95	
			Relevance	13.4	14	Yes	0.95	

Where:

M = Number of items with CVR´values ≥ 0.5823

Acceptable = 95% of the items have CVR´values> 0.5823

Partial CVI´=ΣCVR´/M obtained for the set of items on each aspect assessed (pertinence, relevance and clarity)

Total CVI´= Average of all partial CV

References

Galicia Alarcón, L. A., Balderrama Trápaga, J. A., & Edel Navarro, R. (2017). Validez de contenido por juicio de expertos: propuesta de una herramienta virtual. *Apertura (Guadalajara, Jal.)*, **9**(2), 42-53.

Hyrkäs, K., Appelqvist-Schmidlechner, K., &Oksa, L. (2003). Validating an instrument for clinical supervision using an expert panel. *International Journal of Nursing Studies*, **40**(6), 36-37.

Lawshe, C. H. (1975). A quantitative approach to content validity. *Personnel Psychology,***28**(4), 563–575.

Muñoz-Repiso, A. G. V., Martín, S. C., & Gómez-Pablos, V. B. (2020). Validation of an indicator model (INCODIES) for assessing student digital competence in basic education. *Journal of New Approaches in Educational Research (NAER Journal)*, **9**(1), 110-125.

Tristán-López, A. (2008). Modificación al modelo de Lawshe para el dictamen cuantitativo de la validez de contenido de un instrumento objetivo. *Avances en Medición*, **6**, 37–48.

ANNEX 2b. Sample size

The sample size was calculated using the standard formula for estimating a proportion in a finite population (Krejcie & Morgan, 1970; Cochran, 1977)::

$$n = \frac{\frac{z^2 x\, p\,(1-p)}{e^2}}{1+\frac{z^2 x\, p\,(1-p)}{e^2 N}}$$

Where:

n: the sample size

N: population size: 43 068 733 hab. (mainland Spain).

e: margin of error (0.05)

p: the proportion of individuals who possess the characteristic being studied (0.5).

q: the proportion of individuals who do not have the study characteristic, that is, it is 1-p.

z: the standard deviation established according to the confidence level

$$N= \frac{\frac{1.96^2 x\ 0.5\ (1\text{-}0.5)}{0.05^2}}{1+\frac{1.96^2\ x\ 0.5\ (1\text{-}0.5)}{0.05^2 x\ 43{,}068{,}733}} = 385$$

Applying these parameters resulted in a minimum required sample size of 385 respondents, ensuring adequate representativeness for a 95% confidence level and a ±5% margin of error. The final sample size of 440 respondents exceeded this requirement, further reinforcing the reliability of our findings.

References:

Cochran, W. G. (1977). Sampling Techniques (3rd ed.). John Wiley & Sons.

Krejcie, R. V., & Morgan, D. W. (1970). Determining Sample Size for Research Activities. Educational and Psychological Measurement, 30(3), 607-610.

ANNEX 3. Respondents' profile

Year of birth	Frequency sample	Percentage sample	Population	Percentage in Spain
1921-1956	101	22.9	9,303,069	24.2
1957-1966	66	15	6,365,192	19.7
1967-1976	83	18.6	7,588,709	18.67
1977-1986	85	19.3	7,181,823	16.5
1987-1996	59	13.6	4,724,266	12.3
1997-2003	46	10.4	3,327,624	8.7
**Educational level**	**Frequency**	**Percentage**		
None	1	0.2	13,700,000	58.6
Primary	24	5.4		
Secondary, high school, technical college	150	34.1		
University students	248	56.4	9,700,000	41.4
Doctorate	17	3.9		
**Association/hobby**	**Frequency**	**Percentage**		
Nature conservation association member	9	2.0		
Work related to the natural environment	10	2.3		
Small game hunter	6	1.4		
Big game hunter	4	0.9		
Farmer	10	2.3		
Rancher	1	0.2		
Owner of land exceeding 50 ha	5	1.1		
None of the above	409	93.0		
Empty	1	0.2		
**Place of residence**	**Frequency**	**Percentage**		
Rural	188	47	22,121,910	46.7
Urban	212	53	25,229,657	53.3
**Sex**	**Frequency**	**Percentage**		
Man	215	49.0	23,206,752	49.0
Woman	225	51.0	24,144,815	51.0
**Autonomous community**	**Frequency**	**Percentage**		
Andalusia	86	19.5	8,464,411	17.9
Aragon	13	2.9	1,329,391	2.8
Asturias	10	2.3	1,018,784	2.2
Balearic Islands	0	0	1,171,543	2.5
Canary Islands	0	0	2,175,952	4.6
Cantabria	4	0.9	582,905	1.2
Castile and León	23	5.2	2,394,918	5.1
Castilla - La Mancha	22	5	2,045,221	4.3
Catalonia	78	17.8	7,780,479	16.4
Valencian Community	48	10.9	5,057,353	10.7
Extremadura	11	2.5	1,063,987	2.3
Galicia	29	6.6	2,701,819	5.7
Madrid	68	15.5	6,779,888	14.3
Murcia	15	3.4	1,511,251	3.2
Navarre	7	1.6	661,197	1.4
Basque Country	23	5.2	2,220,504	4.7
La Rioja	3	0.7	319,914	0.7
Ceuta and Melilla	0	0	171,278	0.4

Annex 4. Fisher exact test of associations between (a) attitudes and (b) behavioural intentions towards management measures and agreement degree. Values indicate Chi^2, and p-value in brackets. Cells in red indicate positive association between attitudes and behaviours and their agreement degree (selected higher than expected). * I would be willing to make a financial donation so that, in my province... ** I would be willing to collaborate as a volunteer through education or awareness actions and report on my social networks in order to... ***I would not contribute in any way to changing this situation because...

a

	Totally agree	Agree	No agree/No disagree	Disagree	Totally disagree	Not sure
**A1. Management hunting**	9.5 (<0.01)	41.2 (<0.01)	8.4 (<0.01)	19.9 (<0.01)	16 (<0.01)	3.8 (0.04)
**A2. Hunter-managed hunting**	0.1 (0.74)	6.1 (<0.01)	1.8 (0.13)	0.4 (0.48)	3.4 (0.04)	1.1 (0.33)
**A3. Recreational hunting**	47.5 (<0.01)	72.2 (<0.01)	0.1 (0.69)	55.4 (<0.01)	154.3 (<0.01)	0.1 (0.81)
**A4. Capture and slaughter**	42.9 (<0.01)	84.3 (<0.01)	0.3 (0.55)	36.9 (<0.01)	205 (<0.01)	2.7 (0.09)
**A5. Translocation**	0.3 (0.55)	2.6 (0.04)	0.0 (0.9)	1.5 (0.18)	0.7 (0.39)	0.1 (0.71)
**A6. Contraception**	23.5 (<0.01)	3 (0.03)	18.5 (<0.01)	12.9 (<0.01)	3.9 (0.02)	6.5 (0.01)
**A7. Indirect measures**	11.7 (<0.01)	33.9 (<0.01)	0.6 (0.39)	19.9 (<0.01)	37.8 (<0.01)	2.3 (0.11)
**A8. Natural predation**	8.5 (<0.01)	0.7 (0.3)	38.7 (<0.01)	0.6 (0.43)	23.9 (<0.01)	1.4 (0.22)
**A9. Avoid additional resources**	242.1 (<0.01)	4.9 (0.01)	30.2 (<0.01)	46.5 (<0.01)	49.4 (<0.01)	0.7 (0.46)

b

	Totally agree	Agree	No agree/No disagree	Disagree	Totally disagree	Not sure
**B1.*Control programs**	0.0 (0.92)	0.2 (0.56)	3.3 (0.03)	0.4 (0.48)	2.5 (0.07)	0.8 (0.32)
**B2.*Recreational hunting**	13.8 (<0.01)	43.5 (<0.01)	31.6 (<0.01)	2.4 (0.07)	214.7 (<0.01)	4.1 (0.02)
**B3.*Other alternatives**	1.4 (0.24)	5.1 (0.01)	1.6 (0.13)	0.2 (0.7)	0.0 (0.9)	0.2 (0.65)
**B4.*Prevent control programs**	9 (<0.01)	6.2 (<0.01)	5.2 (<0.01)	8.2 (<0.01)	12.1 (<0.01)	0.6 (0.42)
**B5.*Prevent recreational hunting**	86.7 (<0.01)	0.0 (0.95)	5.2 (<0.01)	4.2 (0.02)	0.1 (0.76)	0.8 (0.37)
**B6.*Prevent other alternatives**	6.0 (<0.01)	9.3 (<0.01)	0.1 (0.73)	0.8 (0.31)	6.4 (<0.01)	0.8 (0.32)
**B7.**Population control**	0.1 (0.84)	28.8 (<0.01)	3.2 (0.03)	17.9 (<0.01)	19.6 (<0.01)	5.6 (0.01)
**B8.**Opposition to population control**	13.8 (<0.01)	13.0 (<0.01)	11.3 (<0.01)	1.0 (0.25)	0.9 (0.29)	7.3 (<0.01)
**B9.***Not relevant problem**	6.0 (<0.01)	0.2 (0.56)	30.1 (<0.01)	0.1 (0.75)	20.5 (<0.01)	2.4 (0.11)
**B10.***Others have to take care**	32.4 (<0.01)	46.3 (<0.01)	0.9 (0.26)	7.9 (<0.01)	44.1 (<0.01)	5.6 (<0.01)
**B11.***Natural processes/ No human intervention**	2.9 (0.08)	0.1 (0.75)	2.0 (0.09)	12.4 (<0.01)	30.0 (<0.01)	1.5 (0.21)

Annex 5. Fisher exact test results of associations between (a) attitudes and (b) behavioural intentions towards management measures by socioeconomic context. Values indicate Chi^2, and p-value in brackets. Cells in red indicate positive association between attitudes and behaviours and contexts (selected higher than expected). * I would be willing to make a financial donation so that, in my province... ** I would be willing to collaborate as a volunteer through education or awareness actions and report on my social networks in order to... ***I would not contribute in any way to changing this situation because...

a

	Protected areas	Hunting	Livestock	Forest	Agricultural	Urban
**A1. Management hunting**	15.8 (<0.01)	1.7 (0.11)	8.3 (<0.01)	4.1 (0.01)	5.6 (<0.01)	4.3 (0.01)
**A2. Hunter-managed hunting**	22.1 (<0.01)	158.7 (<0.01)	5.6 (0.01)	0.6 (0.4)	0.0 (0.93)	23.5 (<0.01)
**A3. Recreational hunting**	6.0 (<0.01)	53.1 (<0.01)	0.1 (0.72)	1.5 (0.18)	7.1 (<0.01)	14.1 (<0.01)
**A4. Capture and slaughter**	0.6 (0.46)	1.8 (0.18)	5.8 (0.01)	1.1 (0.23)	0.1 (0.88)	1.1 (0.3)
**A5. Translocation**	0.0 (1.0)	4.4 (0.01)	1.6 (0.13)	0.0 (1.00)	0.5 (0.4)	0.0 (0.89)
**A6. Contraception**	15.6 (<0.01)	1.6 (0.18)	0.3 (0.54)	1.6 (0.15)	5.5 (0.01)	4.6 (0.01)
**A7. Indirect measures**	8.3 (<0.01)	45.6 (<0.01)	64.7 (<0.01)	12.8 (<0.01)	123.2 (<0.01)	35.2 (<0.01)
**A8. Natural predation**	28.5 (<0.01)	1.1 (0.27)	5.0 (0.01)	30.7 (<0.01)	9.1 (<0.01)	20.8 (<0.01)
**A9. Avoid additional resources**	5.1 (0.01)	27.7 (<0.01)	23.7 (<0.01)	14.6 (<0.01)	22.5 (<0.01)	439.2 (<0.01)

b

	Protected areas	Hunting	Livestock	Forest	Agricultural	Urban
**B1.*Control programs**	0.1 (0.75)	13.2 (<0.01)	1.0 (0.25)	1.5 (0.14)	0.8 (0.27)	5.1 (<0.01)
**B2.*Recreational hunting**	7.0 (<0.01)	47.9 (<0.01)	1.8 (0.14)	0.0 (0.81)	0.2 (0.63)	12.3 (<0.01)
**B3.*Other alternatives**	3.1 (<0.01)	7.3 (0.01)	15.4 (<0.01)	0.1 (0.79)	4.8 (0.01)	1.7 (0.15)
**B4.*Prevent control programs**	0.0 (1.00)	1.2 (0.33)	3.4 (0.05)	2.4 (0.10)	0.0 (0.87)	5.0 (<0.01)
**B5.*Prevent recreational hunting**	0.6 (0.37)	38.9 (<0.01)	3.0 (0.06)	0.4 (0.52)	3.5 (0.03)	8.1 (<0.01)
**B6.*Prevent other alternatives**	1.1 (0.25)	3.7 (0.05)	0.2 (0.74)	2.6 (0.07)	1.8 (0.19)	0.2 (0.62)
**B7.**Population control**	0.0 (0.93)	6.1 (<0.01)	0.6 (0.40)	0.1 (0.80)	0.1 (0.74)	3.2 (0.4)
**B8.**Opposition to population control**	0.5 (0.53)	0.1 (0.84)	0.2 (0.67)	0.2 (0.67)	0.2 (0.67)	0.1 (0.84)
**B9.***Not relevant problem**	1.9 (0.13)	0.2 (0.75)	1.9 (0.13)	1.0 (0.33)	0.1 (0.91)	19.2 (<0.01)
**B10. **Others have to take care**	0.1 (0.73)	0.4 (0.45)	0.9 (0.27)	1.0 (0.24)	0.9 (0.27)	0.0 (0.89)
**B11.***Natural processes/ No human intervention**	21.3 (<0.01)	0.0 (1.00)	2.6 (0.08)	6.1 (<0.01)	7.8 (<0.01)	6.8 (<0.01)
**B12. ***Other reasons**	0.1 (0.76)	0.1 (0.84)	0.0 (1.00)	0.4 (0.54)	0.2 (0.68)	1.8 (0.15)

ANNEX 6. Global relationships between behavioural intentions and management attitudes by socioeconomic contexts. Attitudes: 1. Management hunting; 2. Hunter-managed hunting; 3. Recreational hunting; 4. Capture and slaughter; 5. Translocation; 6. Contraception. 7; Indirect measures; 8. Natural predation; 9. Avoid additional resources. * I would be willing to make a financial donation so that... ** I would be willing to collaborate as a volunteer through education or awareness actions and report on my social networks in order to... ***I would not contribute in any way to changing this situation because... Behavioural intentions: 1.*Control programs; 2.*Recreational hunting; 3.*Other alternatives; 4.*Prevent control programs; 5.*Prevent recreational hunting; 6.*Prevent other alternatives; 7.**Population control; 8.**Opposition to population control; 9.***Not relevant problem; 10.***Others have to take care; 11.***Natural processes/No human intervention; 12***Other reasons.

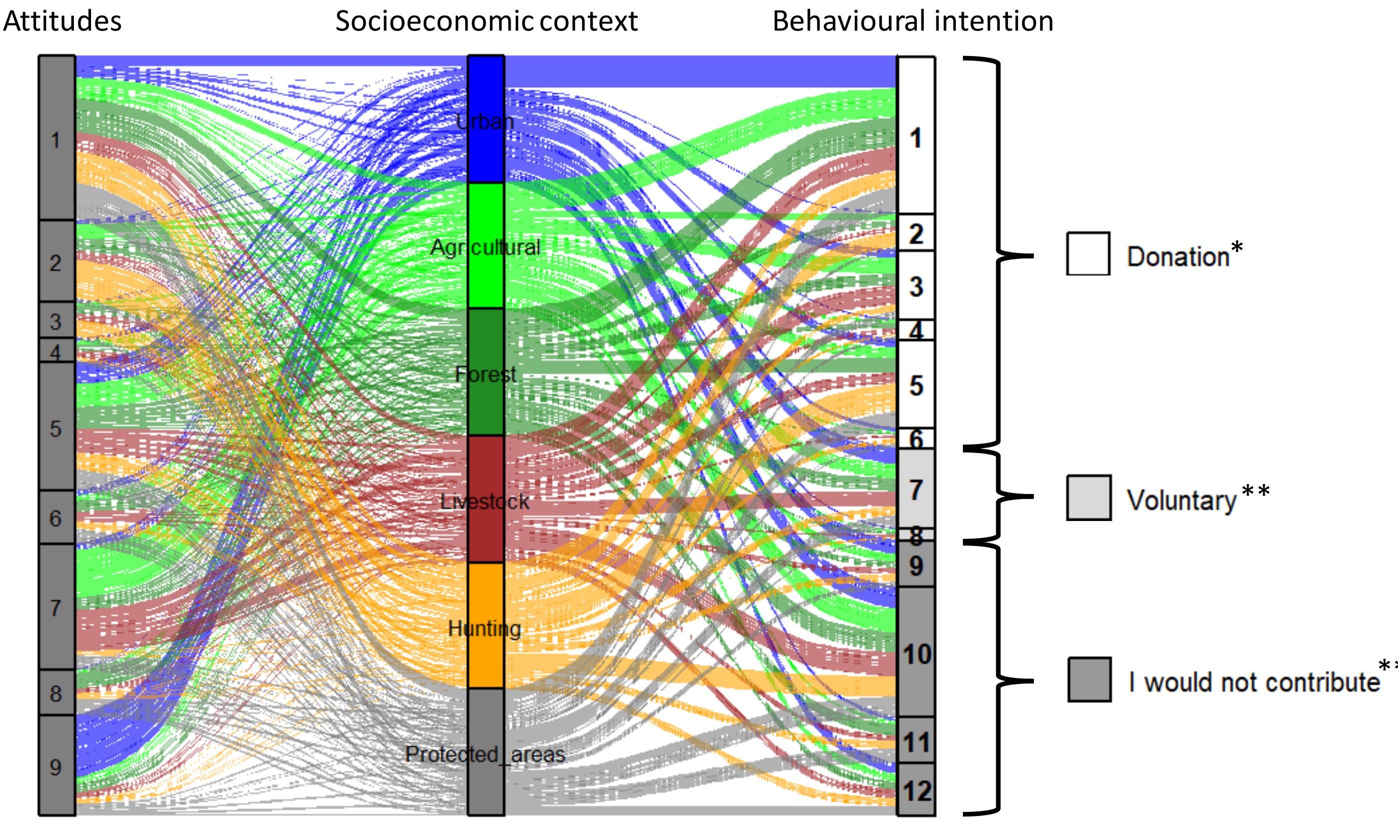

Supplement: Supplementary file 1 — Supplementary file1 (PDF 587 KB) [file 13280_2025_2258_MOESM1_ESM.pdf]
